# Revisiting: “prevalence of and factors associated with sarcopenia among multi-ethnic ambulatory older Asians with type 2 diabetes mellitus in a primary care setting”

**DOI:** 10.1186/s12877-020-01727-0

**Published:** 2020-10-20

**Authors:** Xueting Li, Fengqiang Xu, Longgang Hu, Hao Fang, Yi An

**Affiliations:** 1 Heart Center and Beijing Key Laboratory of Hypertension, Beijing Chaoyang Hospital, Capital Medical University, Beijing, 100020 China; 2 Medical Research Center, Beijing Chaoyang Hospital, Capital Medical University, Beijing, 100020 China; 3 Department of Cardiology, The Affiliated Hospital of Qingdao University, No.1677 Wutaishan Road, Huangdao District, Qingdao, 266000 China; 4 Department of Cardiology, The Affiliated Cardiovascular Hospital of Qingdao University, Qingdao, 266000 China; 5 Department of Cardiology, Rizhao People’s Hospital, Rizhao, 276800 China

**Keywords:** Sarcopenia, Diabetes, COPD

## Abstract

**Background:**

Sarcopenia is an age-related clinical syndrome characterized by loss of muscle mass and reduced muscle function. Diseases that contribute to sarcopenia include type 2 diabetes mellitus (T2DM), chronic obstructive pulmonary disease (COPD), heart failure, chronic kidney disease, and cancer and others. Fung FY et al. (BMC Geriatrics. 2019;19(1):122) conducted a single-center study aimed to determine the prevalence of sarcopenia among older patients with T2DM and to identify factors which mitigate sarcopenia. Their study entitled *“Prevalence of and factors associated with sarcopenia among multi-ethnic ambulatory older Asians with type 2 diabetes mellitus in a primary care setting”* suggested that the prevalence of sarcopenia in older patients with T2DM was 27.4%, and that Chinese ethnicity was associated with a greater risk of sarcopenia in the study population.

**Discussion:**

Deficiency in scientific research and analysis of other diseases associated with sarcopenia such as COPD, may contribute to misestimation of the prevalence of sarcopenia in older patients with T2DM. We are concerned that the conclusions of this single-center study with a small study population might be unreliable.

**Summary:**

The prevalence of sarcopenia in older patients with T2DM in a single-center study with a small sample size may be misestimated due to the lack of strict exclusion criteria and detailed analysis of other diseases that contribute to sarcopenia. In addition, it is inappropriate to draw the conclusion that Chinese ethnic group was associated with a greater risk of sarcopenia among the study population.

## Main text

We read the article *“Prevalence of and factors associated with sarcopenia among multi-ethnic ambulatory older Asians with type 2 diabetes mellitus in a primary care setting”* with great interest [1]. This study reported that the prevalence of sarcopenia in unassisted ambulatory older, community-dwelling patients with type 2 diabetes mellitus (T2DM) in Singapore was 27.4%, and that Chinese ethnicity was associated with a greater risk of sarcopenia among the study population.

Chronic obstructive pulmonary disease (COPD) can lead to malnutrition, weight loss and induce the development of sarcopenia [2]. The prevalence of sarcopenia in patients with COPD is up to 15–40% [3–6]. In addition to COPD and T2DM, sarcopenia has previously been reported to be associated with various diseases, such as chronic heart failure, chronic kidney disease and cancer. We cannot find the strict exclusion or detailed analysis of the diseases related to sarcopenia in this study. Since this was a single-center research with a small study population, the prevalence of sarcopenia in older patients with T2DM may be largely affected by other diseases such as COPD, among whom the prevalence of sarcopenia is also considerable. Did the authors analyze whether the patients in the sarcopenia and severe sarcopenia groups have complicating COPD or other diseases contributing to sarcopenia? Furthermore, the morbidities of sarcopenia in different ethnicities may be influenced by many factors, and it is inappropriate to draw the conclusion that Chinese ethnic groups were associated with a greater risk of sarcopenia among older patients with T2DM. For example, genetic background plays an important role in the development of COPD, and the morbidity of COPD differs in ethnicities. Did the authors analyze whether the prevalence of COPD in patients of Chinese ethnicity was higher than that in other ethnicities among the study population? Inappropriate and unscientific research design can lead to unreliable results. Did Fung FY et al. take all these questions into consideration? The ignorance of these above issues could affect the results and interpretation of the study. Further studies and analyses should be performed taking into consideration the concerns we have mentioned.

## Data Availability

Not applicable.

## Abbreviations

T2DM: Type 2 diabetes mellitus
COPD: Chronic obstructive pulmonary disease
